# Ultrastructural evidence for nutritional relationships between a marine colonial invertebrate (Bryozoa) and its bacterial symbionts

**DOI:** 10.1007/s13199-017-0516-1

**Published:** 2017-11-03

**Authors:** N. P. Karagodina, A. E. Vishnyakov, O. N. Kotenko, A. L. Maltseva, A. N. Ostrovsky

**Affiliations:** 1 Department of Invertebrate Zoology, Faculty of Biology, Saint Petersburg State University, Universitetskaja nab. 7/9, 199034 Saint Petersburg, Russia; 2 Department of Palaeontology, Faculty of Earth Sciences, Geography and Astronomy, Geozentrum, University of Vienna, Althanstrasse 14, A-1090 Vienna, Austria

**Keywords:** Bryozoa, Bacteria, Symbiosis, Ultrastructure, Nourishment

## Abstract

Autozooids of the cheilostome bryozoan *Aquiloniella scabra* contain rod-like bacteria in the funicular bodies – the complex swellings of the funicular strands. Each funicular body contains symbionts in the central cavity surrounded by a large, synthetically active internal “sheath-cell” (bacteriocyte) and a group of the flat external cells. The tightly interdigitating lobes of these cells form a capsule well-isolated from the body cavity. Slit-like spaces between bacteria are filled with electron-dense matrix and cytoplasmic processes of various sizes and shapes (often branching) produced by the “sheath-cell”. The cell ultrastructure and complex construction of the funicular bodies as well as multiplication of the bacteria in them suggest metabolic exchange between host and symbiont, involving the nourishment of bacteria. We suggest that the bacteria, in turn, influence the bryozoan mesothelial tissue to form the funicular bodies as capsules for bacterial incubation. We present ultrastructural data, discuss possible variants in the development of the funicular bodies in Bryozoa, and propose the possible role of bacteria in the life of their bryozoan host.

## Introduction

Symbiotic (and, particularly, mutualistic) co-existence implies a bidirectional exchange of substances between the members of the system (Schmidt 2008). Long-term presence and, as a rule, reproduction of symbionts populating the host body inevitably rely on the transport of gases and nourishment provided by the host. The physiological mechanisms and morphological adaptations behind these processes are often unknown or poorly studied. In aquatic modular invertebrates, symbiotic systems such as corals+zooxantellae and sponges+bacteria have received much attention (reviewed in Rowan 1998; Baker 2003; Taylor et al. 2007; Vishnyakov and Ereskovsky 2009; Webster et al. 2010). Almost nothing, however, is known about symbionts of Bryozoa in this respect.

Together with sponges and cnidarians, bryozoans are among the dominant epibiotic groups in most benthic communities (McKinney and Jackson 1989; Ryland 2005). They are filter-feeders predominantly consuming unicellular algae (Winston 1977; Shunatova and Ostrovsky 2001). Each bryozoan colony consists of the structurally and physiologically interconnected feeding modules (autozooids). The latter are represented by a body wall enveloping a coelomic body cavity (cystid) and a retractile crown of ciliary tentacles and a U-shaped gut (polypide) with ganglion, nerves and accompanying musculature. Zooidal coeloms are interconnected via communication pores that in cheilostome bryozoans are associated with mesothelial funicular cords. These cords connect gut and pores in the cystid wall thus performing a transport function inside and between zooids (Carle and Ruppert 1983; Lutaud 1985). The life cycle includes a swimming larva metamorphosing to the first autozooid (ancestrula), initiating an iterative zooidal budding to form a colony (Reed 1991; Mukai et al. 1997).

Symbionts were discovered in Bryozoa by Lutaud (1964, 1965). Initially, bacteria-like bodies were detected in so-called vestibular organs connected with the zooidal aperture in autozooids and avicularia (polymorphic zooids). They were subsequently found in so-called funicular bodies (Lutaud 1969; Dyrynda and King 1982; Zimmer and Woollacott 1983; Boyle et al. 1987; Mathew et al. accepted) and lacunae of the funicular strands (Woollacott and Zimmer 1975). In all cases, bacteria are extracellular, except in one species where they are also encountered inside pharyngeal cells (Lutaud 1986). Importantly, until now symbionts were exclusively found in Cheilostomata – the youngest, largest and morphologically most diverse bryozoan clade (Taylor and Waeschenbach 2015). All species possessing symbionts belong to eight distant families, suggesting independent acquisition of bacteria, which also differ in appearance.

Except for Lutaud (1986), who applied transmission electron microscopy (TEM), all the above data were obtained using light microscopy. About the same time, bacteria were found by TEM in the bryozoan larvae (Woollacott 1981; Zimmer and Woollacott 1983, 1989; Boyle et al. 1987). This suggests a vertical transmission of the symbionts. Rod- and irregular-shaped bacteria were detected inside a ring-like groove (pallial sinus) of the larvae in a few species of the genus *Bugula* and inside the supracoronal groove in the larvae of *Watersipora*. These findings as well as the discovery of bryostatin 1 (cyclic macrolide with a strong antileukemic activity) in *Bugula neritina* (Pettit et al. 1982; reviewed in Trindade-Silva et al. 2010) triggered extensive research on the model system consisting of this bryozoan species and its bacterial symbiont, which was finally identified as a gram-negative γ–proteobacteria called “*Candidatus* Endobugula sertula” (Haygood and Davidson 1997). Further studies demonstrated the biosynthesis of bryostatins by these bacteria (Davidson et al. 2001). Moreover, experiments showed that larvae of *B. neritina* are unpalatable for fish predators, being chemically protected by bryostatin 20 produced by the symbiont (Lindquist and Hay 1996; Lopanik et al. 2004a, b). Further studies proved the vertical transmission of the uncultured symbiont in the life-cycle of *B. neritina* (Sharp et al. 2007a). The same method was suggested for the symbionts of two species from the genus *Watersipora* identified as gram-negative α–proteobacteria “*Candidatus* Endowatersipora palomitas” and “*C.* Endowatersipora rubus” correspondingly (Anderson and Haygood 2007). Another gram-negative γ–proteobacteria “*Candidatus* Endobugula glebosa” and its metabolites (similar to bryostatins) have been found in the larvae of *Bugulina simplex* (Lim and Haygood 2004). Further, presence of the related symbionts was recorded detecting 16S SSU rRNA gene sequences in two more bugulid species (Lim-Fong et al. 2008). Recently, bacteria were reported in the funicular strands and a brood chamber with larva in another confamiliar cheilostome based on TEM (Moosbrugger et al. 2012).

The role of the symbionts in the life of the adult bryozoan hosts is unknown. It was widely suggested that the substances they produce have antifouling properties (see, for instance, Shellenberger and Ross 1998; Paul et al. 2007; Sharp et al. 2007b, 2008). Experiments also showed that colonies grown from the larvae treated with antibiotics (and thus devoid of bacteria) show much lower fecundity (produce far fewer larvae) (Mathew et al. 2016), indicating a strong dependence of oogenesis on the bacterial metabolites.

Nonetheless, the morphological and physiological relationships between bryozoans and their bacterial symbionts are known so poorly that the nature of the interactions in the different stages of their life cycles remained open to speculation. Among the main questions are how bacteria interact with the host tissues, how they move inside them and how they are transmitted to the brooded larva. While molecular studies currently dominate in this field, one of the efficient methods to better understand the intimate relations between the host and its symbionts is TEM. Only three papers include scant information on the ultrastructural relationships between bacteria and bryozoan tissues (Woollacott and Zimmer 1975; Zimmer and Woollacott 1983; Lutaud 1986). This paper presents the first ultrastructural data on bacteria in the bryozoan funicular bodies. For this ongoing study we selected the cheilostome bryozoan *Aquiloniella scabra*, whose relative (*Scrupocellaria scruposa*) was already reported as having bacteria in the funicular bodies (Lutaud 1969). No TEM-research has been ever done on these special structures. Intriguingly, we provide evidence of a nutritional relationship between the host and the symbiotic cells, suggesting an active role of bacteria towards bryozoan tissues.

## Materials and methods

Colonies of the common boreal-arctic cheilostome bryozoan *Aquiloniella scabra* (van Beneden 1848) (Candidae) were collected from brown algae by SCUBA at 5 m depth near Matrenin Island (Chupa Inlet, Kandalaksha Bay, White Sea), in a close proximity from the Educational and Research Station “Belomorskaia”, Saint Petersburg State University.

**Fig. 1:**
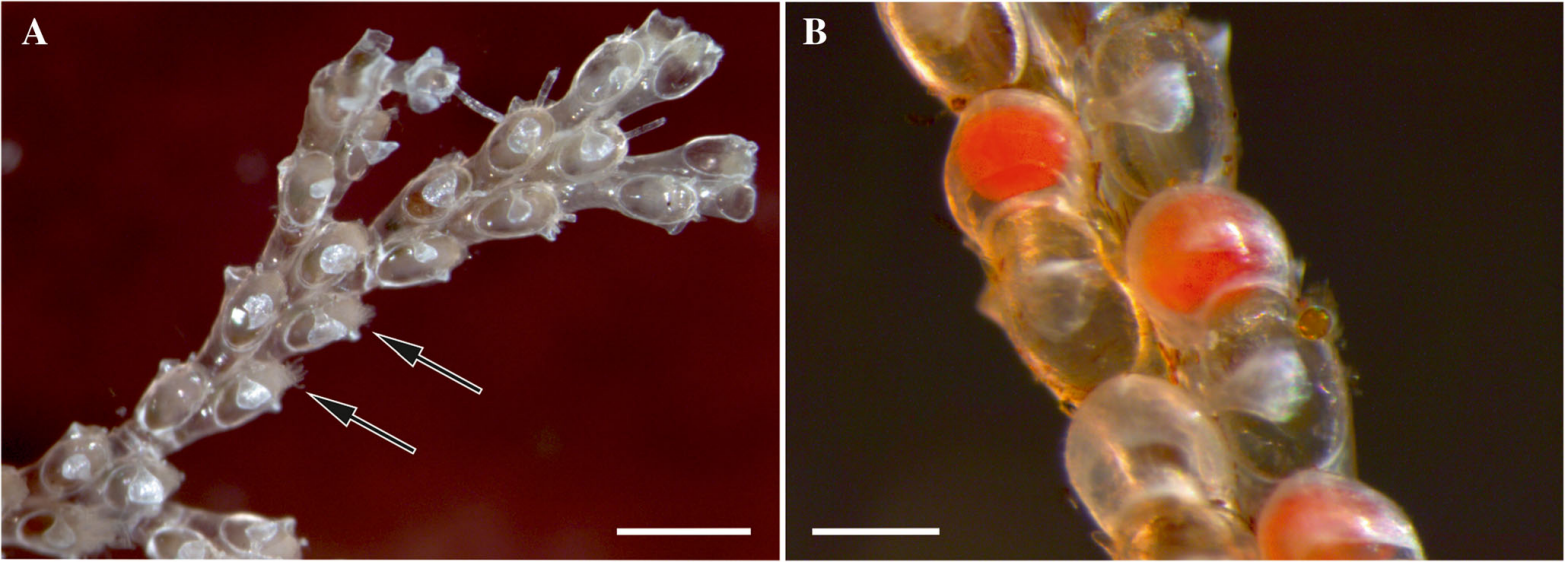
Optical macrophotographs of *Aquiloniella scabra*. **a**, distal part of the living colony fragment with three fully-formed ramifications and two autozooids with partially retracted tentacle crowns (shown by arrows); **b**, part of the branch showing autozooids with retracted polypide and embryos inside brood chambers. Scale bars: **a**, 500 μm, **b**, 200 μm

Weakly-calcified erect colonies of *A. scabra* consist of bifurcating branches (Fig. 1a) being attached to the algal substrate by rhizoid-like polymorphs (kenozooids). Each branch is a paired row of elongated autozooids (sterile or hermaphrodite) with a retractile tentacle crown. Hermaphrodite zooids are associated with the helmet-like brood chambers (ovicells) bearing embryos during reproductive season (Fig. 1b). Ciliated lecitotrophic larvae are short-living, produced in spring and summer.

Specimens were fixed in 2.5% glutaraldehyde (buffered in 0.1 M Na-cacodylate with 10.26% sucrose, pH 7.4) for 1–2 h and subsequently rinsed three times in the buffer. Colonies were sectioned into small fragments and postfixed with a 1–2% solution of osmium tetroxide (OsO4) during 1 h followed by three rinses, each lasting 20 min, in distilled water. Decalcification was conducted for 24 h in 2% aqueous solution of EDTA. After three steps of 10 min washing with distilled water, branches were dehydrated in a graded ethanol series (30-50-70-80-90-100%) and subsequently embedded in epoxy resin type TAAB 812 and sectioned (70 nm thick). To find the area for the TEM study, histological sections (1.0 μm thick) were prepared for light microscopy and stained with Richardson’s stain using standard methods. Ultrathin sections were picked up with copper grids (200 and 300 mesh-size) and contrasted with uranyl acetate and lead citrate. Semithin sections were studied under a Zeiss Axio Imager.A1 light microscope. Ultrathin sections were examined using the transmission electron microscope JEOL JEM-2100HC and photographed with a digital CCD camera.

## Results

### Ultrastructure of funicular bodies

In autozooid the retracted polypide occupies the most part of its coelomic space (Fig. 1a) being surrounded by a network of thin branching and anastomozing funicular cords. These cords (strands) begin on the gut surface further expanding towards the cystid wall and its communication pores. Funicular bodies with bacteria have been found in most studied zooids. It is not clear, however, if all zooids in a colony have them.

**Fig. 2:**
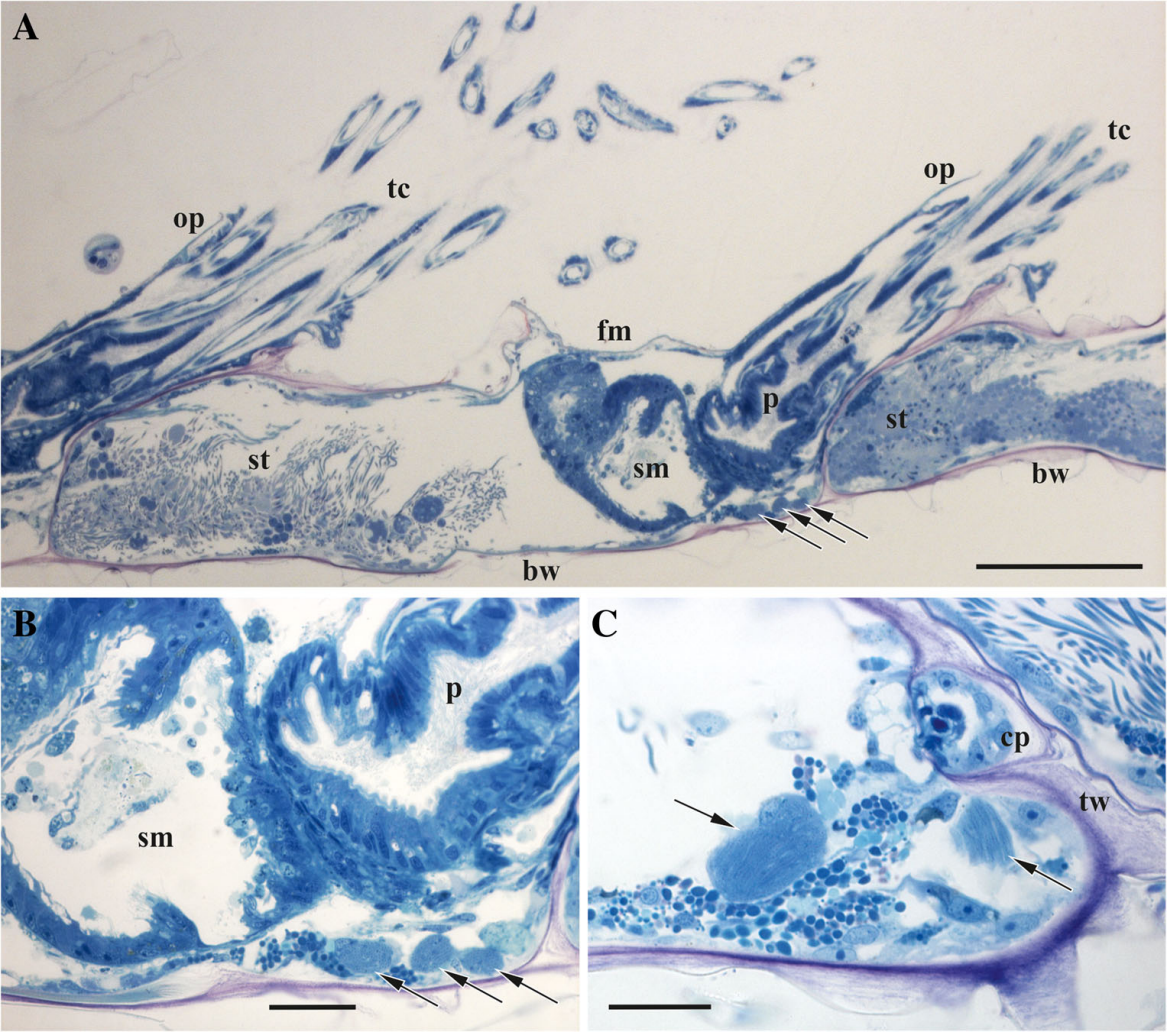
Hystological sections of *Aquiloniella scabra*. **a**, longitudinal section of the colony branch showing three autozooids in row (two with partially protruded polypide and expanded tentacle crowns; proximal and distal zooids shown only partially). Central zooid with three funicular bodies on basal zooidal wall beneath the digestive tract. **b**, The same funicular bodies of various sizes (shown in slightly different section plane). **c**, Large funicular body near transverse zooidal wall with communication pore (second funicular body visible only partially). Funicular bodies shown by arrows. Depending on orientation, the bacteria seen as dark dots or striation. Abbreviations: bw, basal zooidal wall; cp, communication pore; fm, frontal membrane; op, operculum; p, pharynx; sm, stomach; st, spermatogenic tissue; tc, tentacle crown; tw, transverse zooidal wall. Scale bars: **a**, 100 μm, **b**, **c**, 20 μm

**Fig. 3:**
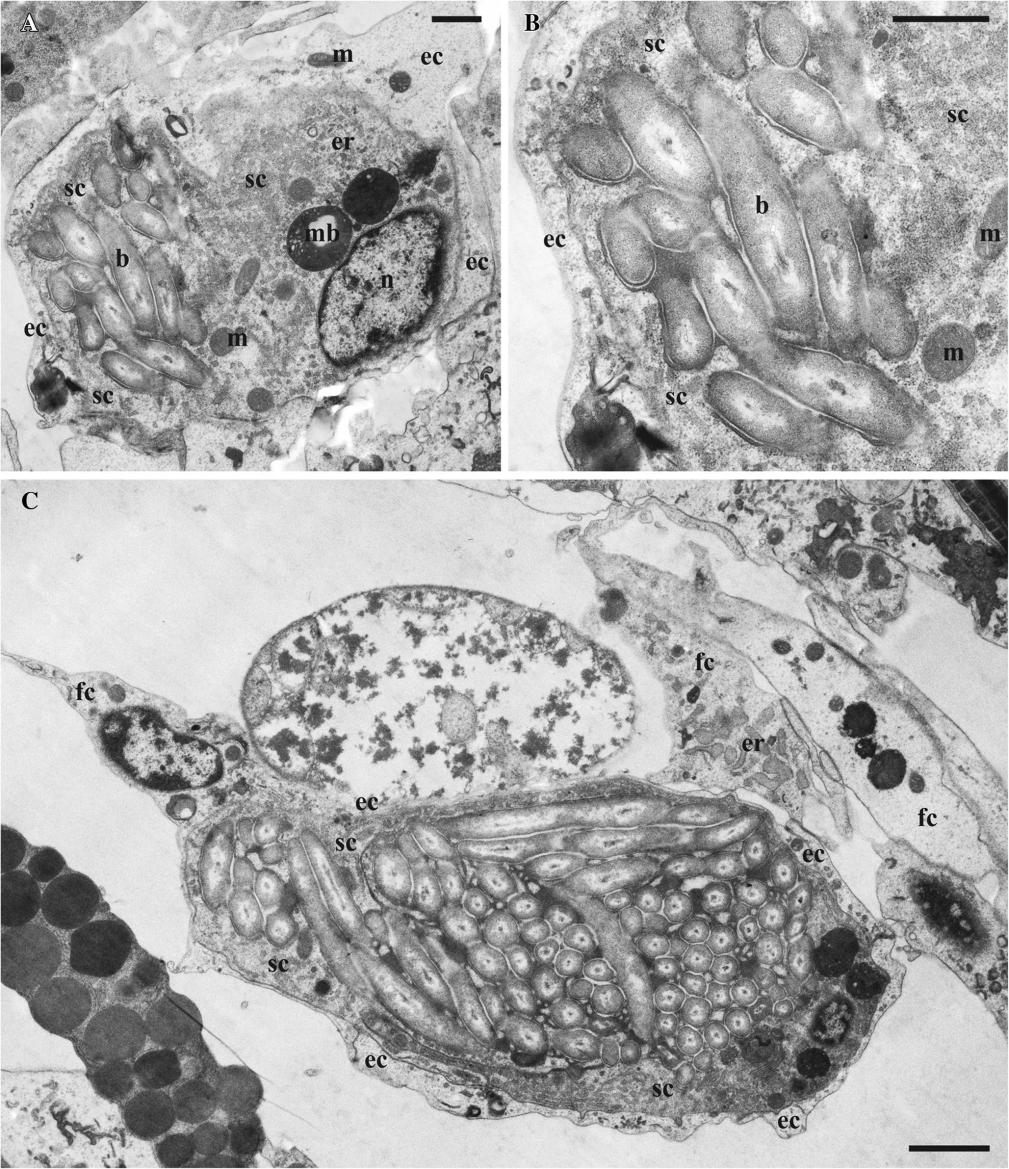
Ultrastructure of the funicular bodies in *Aquiloniella scabra*. **a**, **b**, small funicular body with few symbionts in bacteriophorous vacuoles; **c**, large funicular body with bacteria in the central cavity. Abbreviations: b, bacteria; ec, external cell; er, endoplasmic reticulum; fc, funicular cell; m, mitochondria; mb, multivesicular body; n, nucleus; sc, “sheath-cell”. Scale bars: **a**, **b**, 1 μm, **c**, 2 μm

Funicular bodies are round or oval (sometimes elongated) swellings of the funicular strands predominantly found in the distal half of autozooid either on the basal zooidal wall beneath the gut (Fig. 2), or suspended in the coelomic cavity close to the lateral or frontal walls. We have found smaller (8.1×6.7 μm) as well as larger (21.6×13.5 μm) bodies with few or many bacteria, respectively, possibly corresponding to different developmental stages (Figs. 2 and 3). The size range of 13 funicular bodies found was 6.7–13.3×8.1–24.6 μm. From one to four bodies of various sizes were recorded in one autozooid.

Each mature funicular body is a cellular capsule with a central cavity filled with symbionts (Fig. 3). In our sections, bacteria were enveloped by one large ‘internal’ cell forming a sac-like sheath around them. Since this “sheath-cell” (bacteriocyte) is curved/convoluted, its opposite edges meet, forming interdigitating, lobe-like processes with tight contacts in between.

**Fig. 4:**
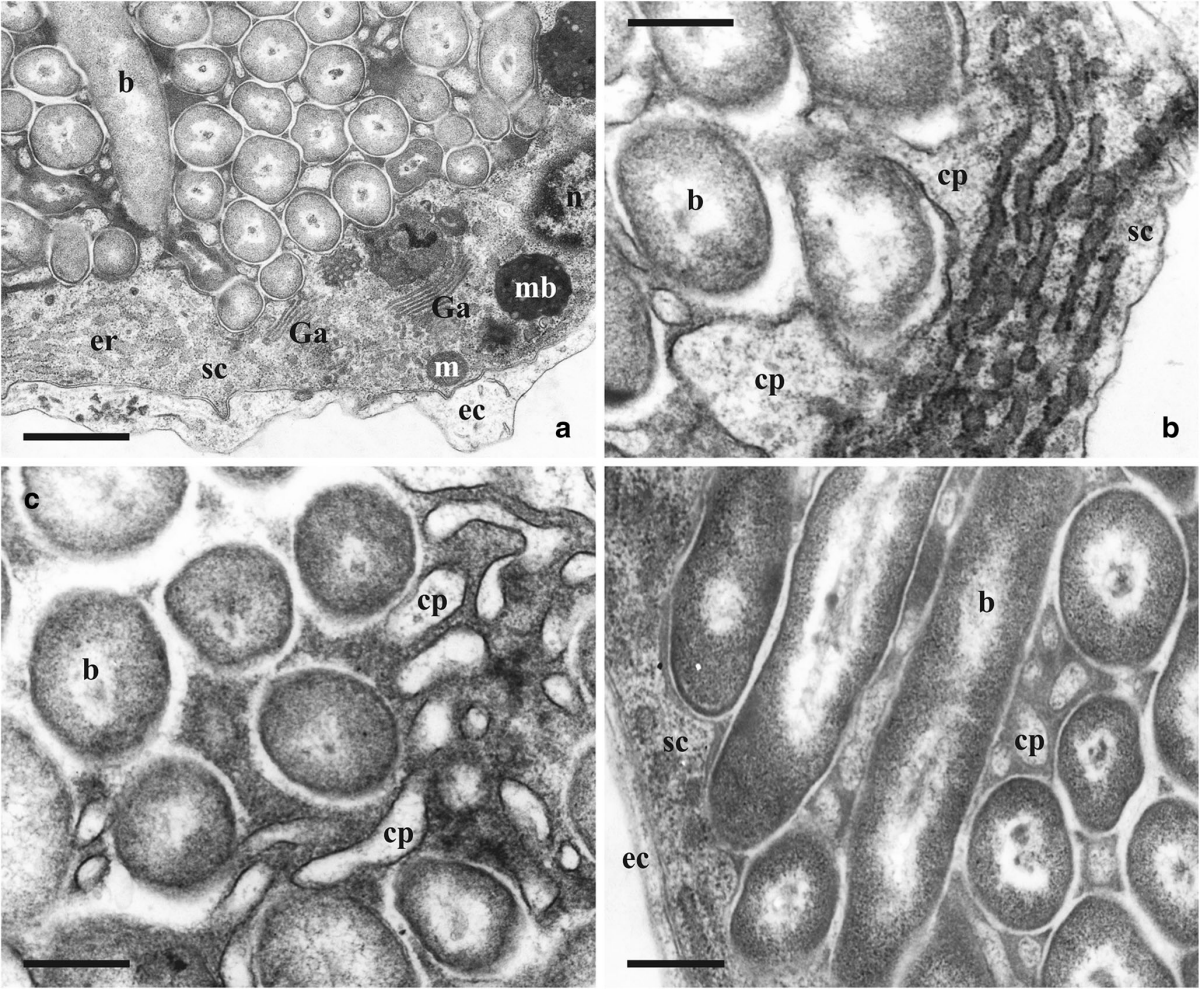
Details of the funicular bodies in *Aquiloniella scabra*. **a**, part of the funicular body showing bacteria enveloped by the electron-dense “sheath-cell” (bacteriocyte) and electron-transparent external cells; **b**, part of the “sheath-cell” with rough endoplasmatic reticulum and two cytoplasmic processes entering spaces between bacteria. External cells are absent in this area; **c**, **d**, bacteria interspersed by the cytoplasmic processes (some branching) of the “sheath-cell” and immersed to the electron-dense matrix. Abbreviations: b, bacteria; cp, cytoplasmic process, ec, external cell; er, endoplasmic reticulum; Ga, Golgi apparatus, m, mitochondria; mb, multivesicular body; n, nucleus; sc, “sheath-cell”. Scale bars: **a**, 1 μm, **b**–**d**, 500 nm

The “sheath-cell” has a large active nucleus with euchromatin occupying most of its volume, and an electron-dense (dark) cytoplasm containing mitochondria, Golgi-apparatus(es), numerous free ribosomes and well-developed rough endoplasmic reticulum (ER) with flattened as well as vesicular cisternae (Figs. 3c and 4a, b). Various vesicular inclusions and, sometimes, electron-dense multivesicular bodies were encountered in the cytoplasm too. Another prominent feature was the numerous cytoplasmic processes on the internal (facing the symbionts) side of the bacteriocyte, entering the slit-like spaces between the bacterial cells (Fig. 4b–d). These processes are round or oval in cross-section and strongly differ in their diameter and length (up to 10 times). They also often branch (Fig. 4c). Thus, the narrow spaces between symbiotic cells are filled with these processes together with electron-dense matrix (Fig. 4). In the young funicular bodies containing few bacteria, the latter are simply embedded in the cytoplasm of the “sheath-cell”, being inside the bacteriophorous vacuoles (Fig. 3b).

Externally the bacteriocyte is overlaid by 1–2 layers of flattened cells with electron-transparent cytoplasm and fewer organelles (Figs. 3b, c and 4a, d). The adjoining edges of these ‘light’ cells often overlap. In some instances they form interdigitating lobe-like processes, in which case up to five layers were visible. The nuclei of the external cells were active with euchromatin filling in most part of their volume.

In one instance, a part of the bacteriocyte was not covered by the external cells and faced the body cavity (Fig. 4b). Also, a lobe-like process of the ‘light’ external cell was seen to partially surround bacteria in one funicular body. The dominating structural role of the “sheath-cell”, however, is certain.

Since the funicular body is a swelling of the funicular strand, the cells of the latter participate in the formation of the external layer of the funicular body, usually at two opposite ‘poles’. In some cases, the external cells of the capsule form processes that are incorporated into the funicular strand (Fig. 3c).

### Bacterial symbionts

Bacterial cells reached 0.5×0.7 μm in diameter and up to 10.0 μm in length in our sections. The central part of the cell is occupied by nucleoid filaments surrounded by an electron-transparent zone. The peripheral cytoplasm is electron-dense, granular, without inclusions, surrounded by a well-recognizable plasma membrane. The wide, electron-transparent zone (that is possibly a periplasmic space) surrounding the cell often fuses with a similar zone of the neighbouring bacteria (Figs. 3 and 4). Sometimes membrane-like fragments are visible in this zone, and, in case if bacteria are gram-negative, they could be remnants of the external membrane of the cell wall. More work is required to clarify this issue.

Depending of the size (age?) of the bacterial body, the relative position of the bacterial cells differed. It was more chaotic in the smaller funicular bodies containing 10–15 symbionts (Fig. 3a, b). In the larger and fully-formed funicular bodies the number of bacteria varied from 70 to 80 to about 130, and their position was more regular. Most cells were in groups positioned parallel to each other, although some had a perpendicular position (Fig. 3c). Note that the above numbers are underestimates because we could count only those symbionts visible in a section plane.

## Discussion

### Structure and origin of the funicular bodies

The structure of the funicular bodies in *Aquiloniella scabra* differs from what was described earlier in certain other cheilostomes (Lutaud 1969; Mathew et al. accepted) having a wall of two layers, namely an internal layer of prismatic, cubic or flattened cells and an external one of flattened cells. According to Lutaud (1969) the wall structure of the funicular body corresponds to that of the zooidal body wall, being represented by epithelial (internal) and perithoneal (external) cell layers. This view is explained by the formation of the funicular body on the body wall and, thus, a direct derivation of its cell layers from those of the zooidal wall.

In the case of *A. scabra,* bacteria are surrounded by the large “sheath-cell” (bacteriocyte) overlaid by the external flattened cells. In our opinion, such a structure suggests that all cells in the funicular body are of mesothelial origin, and no epidermal cells take part in their formation. Although both ‘internal’ and ‘external’ cells are recognizable in the funicular body, it seems more probable, and simpler developmentally, to suggest that its cellular envelope is formed via growth, multiplication and specialization of the funicular cells around bacteria.

We propose the following scenario. After relocation from the larval pallial sinus to the tissues of the ancestrula during metamorphosis (Sharp et al. 2007a), bacteria move to a forming funicular tissue. In the next step, one (several?) bacterial cells immerse into the cytoplasm of the closest funicular cell, which becomes a bacteriocyte. Multiplication of bacteria is accompanied by the corresponding growth of this cell together with the modification of its shape and formation of the central cavity as a result of the enlargement and fusion of the bacteriophorous vacuoles. In the same time, some neighbouring funicular cells multiply and rearrange to form a cellular capsule over the “sheath-cell”. According to this view, only funicular cells of mesothelial origin are involved in the process. This interpretation is supported by the fact that only peritoneal/mesothelial cells constitute the wall of the funicular bodies in two other cheilostomes studied (Dyrynda and King 1982; Zimmer and Woollacott 1983).

Our scenario does not reject the opinion of Lutaud (1969) because both variants are theoretically possible. While observations on the developmental stages of the funicular body are needed to confirm our interpretation, the presence of two variants is in agreement with a mosaic pattern of the bacterial symbiosis distribution in Cheilostomata and its potentially multiple origins in this bryozoan clade.

### Evidence of exchange of substances between host and symbionts

The rod-like bacteria found in *Aquiloniella scabra* are morphologically similar to those earlier recorded in the zooidal tissue and larvae of certain other bryozoans by Lutaud (1986) and Woollacott (1981). We suggest they are gram-negative as in the other cheilostomes in which symbionts were identified using Gram staining (Lutaud 1964, 1969, 1985) and molecular methods (Haygood and Davidson 1997; Lim and Haygood 2004; Anderson and Haygood 2007). In particular, Lutaud (1969) described as gram-negative filamentous bacteria in the funicular bodies of *Scrupocellaria scruposa*, which is closely related to *A. scabra*.

Observed differences in the number of bacteria in the smaller and larger funicular bodies point to bacterial multiplication in *A. scabra*. Similar observations were made by Lutaud (1969) in *Bugulina turbinata*. Clearly, this process requires a gas exchange and nutrient provisioning from the host to bacteria. Removal of their waste metabolites is another important aspect. Lutaud (1986) described symbiotic bacteria in the vestibular organs of the non-related cheilostome *Palmicellaria skenei*, mentioning microvilli formed by the host cells. Although they are isolated from the cavity with bacteria by a thin cuticle, the author suggested these microvilli to absorb the substances produced by bacteria. Else, she suggested the symbionts themselves consume the mucus produced by the glandular diverticulum associated with the vestibular organ. Thus, Lutaud (1986) was the first to suggest an exchange of substances between bryozoans and symbiotic bacteria.

Noteworthy, similar microvillous foldings of the cellular membrane restricted by a cuticle were described in the cells of the placental analogue in two bugulid cheilostomes (Woollacott and Zimmer 1975; Moosbrugger et al. 2012; reviewed in Ostrovsky et al. 2016). In these cases, formation of the foldings was explained by a massive exocytosis during extraembryonic nutrition transporting nutrients across the cuticle to the embryo in the brood cavity. Since membrane foldings and microvilli are known to be involved in both processes – absorption and secretion (Lange 2011; Sauvanet et al. 2015) – both suggestions are plausible. The branching cytoplasmic processes of the “sheath-cell” that are densely interspaced between bacteria in the funicular bodies of *A. scabra* could potentially serve both functions – absorptive and nutritive. These could even be performed simultaneously, with the processes serving as an interface of bidirectional transport between the host and its symbionts. Also, an isolated nature of the intracellular cavity of the bacteria-filled “sheath-cell” could be considered as more effectively promoting an exchange of substances within this system.

Whilst we found no clear ultrastructural indication of exocytosis (although some images suggest similar structures), a well-developed endoplasmic reticulum and numerous ribosomes in the cytoplasm of the bacteriocyte as well as the abundant electron-dense matrix in the slit-like spaces between bacteria and cytoplasmic processes are signs of the synthetic and transport activities performed by the bryozoan. Formation of cytoplasmic processes could be a result of exocytosis – the fusion of exocytic vesicles in this case. In contrast, it is also possible that these processes remain after incomplete fusion of the bacteriophorous vacuoles.

In any case, the complex structure and functioning of the funicular bodies indicate that symbionts actively influence the bryozoan tissue, transforming part of the funicular cord to an “incubator” for their growth and multiplication. The above description and evidence for the exchange of substances (and, thus, reciprocal influence) between the bryozoan host and its bacterial symbionts enables an interesting comparison. While many details differ, the formation and functioning of the funicular bodies in Bryozoa could be compared with the bacteria-induced root nodules known in many higher plants. In both cases the swelling of the transport organ (funicular cord and rootlet correspondingly) occurs via both host and symbiont cell proliferation. In both cases, the symbionts also occupy bacteriophorous vacuoles in the host cells, consuming nutrients and inducing host cell divisions (reviewed in Gage 2004). Although any benefit for the adult host is currently unknown in bryozoans, the strong influence of the bacterial metabolites on host reproduction (and, thus, overall physiology) was recently proven (see Mathew et al. 2016).

As noted above, accumulations of bacteria have also been recorded in the vestibular organs of autozooids and avicularia in several cheilostome species from distant families (Lutaud 1964, 1965, 1969, 1986). While the funicular bodies are presumably derived specializations influenced by symbionts, the vestibular organs may have evolved as organs producing lubricants facilitating excursions of the tentacle crown (see Lutaud 1964; Mukai et al. 1997), and were populated by bacteria later on. In both instances, however, bacteria use the bryozoan tissues/organs as an ‘incubator’ for their nourishment and multiplication.

### Transmission of bacteria to the larvae: pro et contra

We currently do not know if bacteria are transmitted to the larvae of *Aquiloniella scabra*. Presence of the symbionts in both, the adult tissue and larvae was confirmed in two species from the distant families – Bugulidae (Woollacott and Zimmer 1975; Woollacott 1981) and Watersiporidae (Zimmer and Woollacott 1983; Boyle et al. 1987). Further, their transfer between the life-cycle stages was confirmed using immunocytochemical methods in *Bugula neritina* (Sharp et al. 2007a). In the rest of the studied species bacteria were investigated either inside autozooids (Lutaud 1964, 1965, 1969, 1986) or in the larvae (Woollacott 1981; Lim and Haygood 2004; Anderson and Haygood 2007), thus one part of the biphasic life-cycle left unstudied. In the last case, however, it was accepted a priory that symbionts should be present in the adults too because they found in autozooids of related species.

Distribution pattern of bacteria in Bryozoa is very mosaic. While symbionts have been found in the larvae of several species from the genera *Bugulina* and *Crisularia*, they are absent in the larvae of some their congeners (Woollacott 1981; Lim-Fong et al. 2008). Moreover, populations of two sibling species of *Bugula neritina*-complex are infected only partly (McGovern and Hellberg 2003; Linneman et al. 2014). It is doubtfully, however, that bacteria could be present only in the larvae or in the adults. While an opportunity of direct infection of the feeding zooids potentially exists via movement of the swallowed microbe from the gut to the finucular tissue (Linneman et al. 2014), it is unlikely in non-feeding short-living larva. Importantly, till now ‘bryozoan’ bacteria were not identified in the water (Lim-Fong et al. 2008). Thus, whereas an opportunity for horizontal transfer is present when acquiring symbionts anew in a clade where they were previously absent, the entire bulk of evidence supports a view that it is a vertical transfer that maintains the presence of symbionts in the host population (Linneman et al. 2014).

Presence of bacteria inside the lacunae of the funicular cords (Woollacott and Zimmer 1975) in *B. neritina* suggests a route of their transfer from the funicular bodies to the brood chambers containing larvae (Mathew et al. accepted, see also Sharp et al. 2007a). Bacterial cells are aflagellate, and the mode of transport is unknown. Previous histological studies of this species and related cheilostome *Bugulina flagellata* showed presence of the groups of presumed symbionts inside a coelomic cavity of the ooecial vesicle (body wall outgrowth plugging the entrance to the brood chamber) (Mathew et al. accepted, also illustrated in Ostrovsky et al. 2009; Ostrovsky 2013a, b). These observations were confirmed using TEM (Ostrovsky, Schwaha, Moosbrugger, unpublished data) suggesting that after reaching the ovicell via funicular cords, bacteria leave their lacunae and accumulate in small groups before entering the brood cavity. It remains unknown how the symbionts overcome the epithelial wall of the ooecial vesicle covered by a cuticle during a transfer from the coelom to the brooding space.

Based on overall similarity, we assume that the same rout could be used by the symbionts of *A. scabra*. In this case their protective role could be also assumed. This would enable the symbiotic relationship to be more accurately described as mutualistic (Schmidt 2008). However, presence of bacteria in the larvae could not necessarily be an evidence of the presence of defensive substances (Lim-Fong et al. 2008), and experimental evidence is required to prove this.

As to the presence of bacteria in the vestibular organs of some other cheilostome bryozoans, their position close to both the zooidal orifice and entrance to the brood chamber (Lutaud 1964, 1965, 1986) could indicate that bacteria are transmitted to the larva during protrusion of the tentacle crown. They could enter the vestibulum with mucus first, attaching to the tentacles during their passage through the zooidal orifice which is located next to the entrance to the brood chamber.

The function of bacteria living in polymorphic zooids (avicularia) is enigmatic because the latter are not associated with the entrance to the brood chambers (see for instance, Mukai et al. 1997; Lidgard et al. 2012). In case if these symbionts produce toxic metabolites, avicularia could employ them for defensive purposes, but this is unknown.

### Independent origins of bacterial symbiosis in Bryozoa

Secondary metabolites with diverse biological activities have been discovered in a wide range of bryozoans (Christophersen 1985; Anthoni et al. 1990; Prinsep and Morris 1996; Haygood et al. 1999; Sharp et al. 2007b and references therein). The possibility of a symbiotic origin for a number of bryozoan metabolites has been proposed several times, yet has been demonstrated solely for the production of bryostatins in *B. neritina* (Choi et al. 1993; Walls et al. 1995; Davidson et al. 2001; Narkowicz et al. 2002). For instance, bacteria were not detected inside autozooids of the bryozoan *Terminoflustra membranaceotruncata* using light microscopy, whereas brominated substances with strong biological activities were recently described in this species (Maltseva et al. 2014, 2016). Nonetheless, we suggest a very wide distribution of symbiotic bacteria in Bryozoa. Moreover, since bryozoans are structurally and ecologically very diverse, one could additionally hypothesize a variety of the positional and physiological relationships (including nutritional ones) between them and their symbionts. The pattern of distribution of bacteria in distantly related bryozoan taxa, differences in the structure (and, possibly, origin) of the funicular bodies in the studied species, as well as the presence of bacteria from different groups all point to independent origins of this symbiosis. Our current research is focused on these issues and looks very promising.
